# Structural, Spectroscopic, and Dynamic Properties of ${Li}_{2}^{+}(X^{2}\sum_{g}^{+})$ in Interaction with Krypton Atom

**DOI:** 10.3390/molecules28145512

**Published:** 2023-07-19

**Authors:** Samah Saidi, Nesrine Mabrouk, Jamila Dhiflaoui, Hamid Berriche

**Affiliations:** 1 Laboratory of Interfaces and Advanced Materials, Physics Department, Faculty of Sciences of Monastir, Avenue de l’Environnement, Monastir 5019, Tunisiadhiflaouijamila@gmail.com (J.D.); hamid.berriche@aurak.ac.ae (H.B.); 2 Department of Physics, College of Science and Humanities in Al-Kharj, Prince Sattam Bin Abdulaziz University, Al-Kharj 11942, Saudi Arabia; 3 Mathematics and Natural Sciences Department, School of Arts and Sciences, American University of Ras Al Khaimah, Ras Al-Khaimah 10021, United Arab Emirates

**Keywords:** RCCSD(T) method, RKHS interpolation, vibrational quantum bound states, radial and angular distributions

## Abstract

We report a computational study of the potential energy surface (PES) and vibrational bound states for the ground electronic state of ${Li}_{2}^{+}Kr$. The PES was calculated in Jacobi coordinates at the Restricted Coupled Cluster method RCCSD(T) level of calculation and using aug-cc-pVnZ (*n* = 4 and 5) basis sets. Afterward, this PES is extrapolated to the complete basis set (CBS) limit for correction. The obtained interaction energies were, then, interpolated numerically using the reproducing kernel Hilbert space polynomial (RKHS) approach to produce analytic expressions for the 2D-PES. The analytical PES is used to solve the nuclear Schrodinger equation to determine the bound states’ eigenvalues of ${Li}_{2}^{+}Kr$ for a  J = 0 total angular momentum configuration and to understand the effects of orientational anisotropy of the forces and the interplay between the repulsive and attractive interaction within the potential surface. In addition, the radial and angular distributions of some selected bound state levels, which lie below, around, and above the T-shaped 90° barrier well, are calculated and discussed. We note that the radial distributions clearly acquire a more complicated nodal structure and correspond to bending and stretching vibrational motions “mode” of the Kr atom along the radial coordinate, and the situation becomes very different at the highest bound states levels with energies higher than the T-shaped 90° barrier well. The shape of the distributions becomes even more complicated, with extended angular distributions and prominent differences between even and odd states.

## 1. Introduction

Over the past few decades, rare gas media and matrices have attracted great attention as fascinating environments for investigating the spectroscopy, structure, and dynamics embedded inside or deposited on their surfaces. Rare gas atoms are renowned as non-reactive solvents [1,2,3] and provide a model system for both theoretical and experimental investigations involving a large number of atoms, representing a high degree of freedom. Furthermore, as their condensates exhibit transparency across a broad range of radiation energies, inert gas matrices offer the advantage of selectively exciting chromophores embedded within them. Considering the case of alkali atoms, these matrices have proven useful in studying the spectroscopy, photo-association, and optical absorption spectra of alkali atoms [2,4,5]. Matrix isolation spectroscopy (MIS) is a widely utilized technique in various research fields, particularly for investigating alkali atoms in solid matrices of noble gases at cryogenic temperatures [6], as well as studying the high-spin states of alkali molecules [7,8,9,10].

The exceptional characteristics of rare gas atoms, combined with the growing interest in studying alkali atoms following the observation of the photo-association of cold alkali atoms and their Bose–Einstein condensation [4], have made the interaction between alkali atoms and rare gas atoms a prominent research theme in numerous studies. Notably, both experimental and theoretical works [4,11,12,13,14,15,16,17,18,19,20,21,22,23,24,25,26,27,28,29] have been dedicated to exploring the interaction between alkali neutral/ionic dimers and rare gas atoms. The primary focus of these studies is calculating the potential energy surfaces (PES) of dimers with a single rare gas atom. Subsequently, these surfaces are fitted with suitable analytical forms, which are then utilized for dynamic studies such as bound state calculations, as well as for structural investigations, such as the geometric minimization of large-sized clusters to examine the micro-solvation of the embedded dimers.

From an experimental perspective, recent work by Kristensen et al. [21] introduced the use of Coulomb explosion induced by an intense fs laser pulse as a means of studying alkali dimers (${Rb}_{2}$, ${Li}_{2}$, ${Na}_{2}$ and $K_{2}$) on He droplets. They demonstrated that Coulomb explosion enables the determination, within a single measurement, of whether alkali dimers, ${Ak}_{2}$, on the surface of He nanodroplets, are formed in either the $X^{1}\sum_{g}^{+}$ ground state or in the lowest-lying triplet state $a^{3}\sum_{u}^{+}$. Fuchs et al. [19] experimentally investigated the collisional cross-sections of lithium dimer ${Li}_{2}\;$ in selected vibrational states with He and Kr atoms. An der Lan et al. [13] presented high-resolution mass spectra of ${Na}^{+}{He}_{n}$, $K^{+}{He}_{n}$, ${Na}_{2}^{+}{He}_{n}$ and $K_{2}^{+}{He}_{n}$, formed via the electronionization of doped helium droplets. They observed two distinct anomalies in ${Na}_{2}^{+}{He}_{n}$ clusters at *n* = 2 and *n* = 6.

On the theoretical side, Guillon et al. [11] recently performed diffusion and path integral quantum Monte Carlo studies of the rubidium dimer in its ground triplet state in a helium environment [11]. The focus of their work was the influence of helium atoms on the rotational motion of the ${Rb}_{2}$ dimer. Alharzali et al. [12,22], Bodo et al. [17,23,29], and Marinetti et al. [26] focused on the structure and dynamic properties of cationic dimers (${Li}_{2}^{+},\;{Na}_{2}^{+}\;and\;K_{2}^{+})$ interacting with helium atoms using RCCSDT and Post Hartree–Fock approaches. They found that for all three species, the helium atom preferred linear attachment to the cationic dimer rather than a T-shaped configuration. In the case of larger clusters, they observed the solvation of cationic dimers within the helium clusters. Douady et al. [20] investigated the solvation of ionic sodium dimers in argon clusters. They confirmed that the cationic dimer enters the clusters rather than resides at the surface. This behavior is attributed to the relatively strong interaction between the closed-shell neon atoms and the cationic dimer. Zanuttini et al. [15] employed a pseudopotential technique and molecular dynamics with a surface hopping approach to investigate the structure and optical absorption of ${Li}_{2}^{+}$, ${Na}_{2}^{+}$, and $K_{2}^{+}$ alkali dimers in neon clusters. They concluded that the modification of the PES by surrounding neon atoms indicated the strong confinement of the lowest energy states of the three cationic dimers. Saidi et al. [18] explored the structure and stability of the lithium dimer with xenon atoms, while Ghanmi et al. [25] performed a structural, energetic, and spectroscopic study of the potassium cationic dimer interacting with rare gas atoms, such as Ar, Kr, and Xe.

In this paper, we present a computational investigation of the structure and dynamics of the lithium cationic ${Li}_{2}^{+}$ dimer interacting with a Kr atom. Section 3 provides an overview of the computational method details employed in this study, including the basis sets and extrapolation schemes used to calculate the interaction energies of the ${Li}_{2}^{+}Kr$ complex. This potential energy surface is, then, employed in the calculation of bound state levels after being reproduced via the RKHS method [30]. The results are presented and discussed in Section 2. The used methodologies are detailed in Section 3.

## 2. Results and Discussion

The resulting interaction energies were employed to calculate the spectroscopic constants Re  and De for each configuration corresponding to angles ranging from 0° to 90°. The found equilibrium distances (Re) and the well depths (De) for the ${Li}_{2}^{+}Kr$ using the RCCSD(T) method and different basis are summarized in Table 1.

From Table 1, it can be observed that the well depth of the ${Li}_{2}^{+}Kr$ complex at all geometries increases when moving from AVQZ to CBS[Q5]. Taken the example of their collinear arrangement (θ =0°) $D_{e}$ varies from 1730 cm^−1^ to 1737 cm^−1^ for the RCCSD(T)/AVQZ and RCCSD(T)/AV5Z calculations, respectively. However, upon computing the RCCSD(T)/CBS[Q5] using the RCCSD(T)/AVQZ/AV5Z results, the energies increase to 1745 cm^−1^. This indicates that the RCCSD(T)/CBS[Q5] energies are lower than those obtained from the RCCSD(T)/AVQZ and RCCSD(T)/AV5Z estimations.

**Figure 1:**
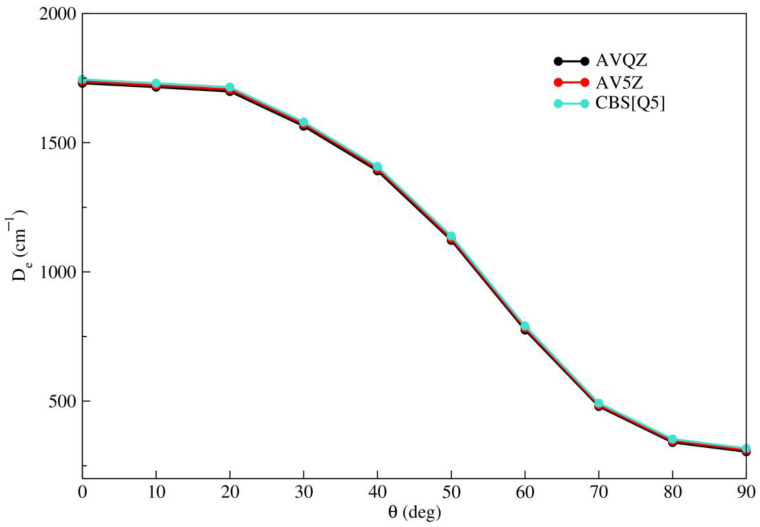
Minimum energy values obtained from RCCSD(T) level of theory using the AVQZ and AV5Z basis sets and CBS extrapolation as a function of θ (deg).

A comparison of the RCCSD(T)/AVQZ, RCCSD(T)/AV5Z, and RCCSD(T)/CBS[Q5] extrapolations is depicted in Figure 1, considering all angular orientations. It can be observed that despite a slight shift between the three curves, the energies obtained from the RCCSD(T)/AVQZ/AV5Z calculations are lower than those from the RCCSD(T)/CBS[Q5] extrapolation. Therefore, the RCCSD(T)/CBS[Q5] energies will be considered as reference data in the subsequent sections.

It is worth noting that as the angle increases from θ = 0° to θ = 90°, the well depth decreases from De = 1745 cm^−1^ to De = 317 cm^−1^. This implies that the linear configuration is the most stable. This arrangement can be explained by considering that the ${Li}_{2}^{+}$ dimer in its ground state can be approximated as two closed-shell *Li*^+^ cores with an electron cloud located between them. Consequently, the short-range repulsion between the electron and the krypton atom, combined with the attraction between the two *Li*^+^ cores and the closed-shell Kr atom, favors the positioning of the krypton atom at the ends of the dimer.

It is important to mention that the interaction of the lithium dimer with noble gas atoms has been extensively studied recently due to its simplicity and significance. Studies conducted by Zanuttini et al. [15] and Alharzali et al. [22] have contributed to this research area. In Table 2, we have grouped the findings from these studies with the current results obtained for ${Li}_{2}^{+}Kr$. It is evident that the repulsive interactions decrease in their impact compared to the attractive long-range interaction forces when moving from ${Li}_{2}^{+}He$ to ${Li}_{2}^{+}Kr$. In fact, the well depths in their linear configuration increase from 380 cm^−1^ for ${Li}_{2}^{+}He$ to 700 cm^−1^ for ${Li}_{2}^{+}Ne$ and further to 1745 cm^−1^ for ${Li}_{2}^{+}Kr$. Hence, the interactions of the three rare gas atoms (He, Ne, Kr) with the lithium dimer become increasingly more attractive, exhibiting larger attractive cores as one moves from helium to krypton.

**Figure 2:**
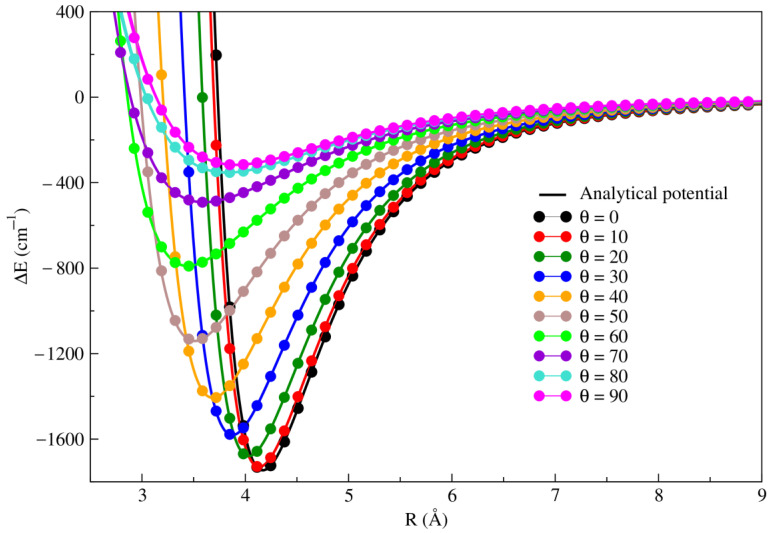
Comparison between the 2D-PES RKHS analytical fitting and the RCCSD(T)/CBS(Q5) ab-initio surfaces of ${Li}_{2}^{+}Kr$.

The RKHS potential curves together with the ab-initio RCCSD(T)/CBS[Q5] interaction energies along the R coordinate for each θ angle from 0° to 90° are illustrated in Figure 2. A good agreement is observed between the RKHS potential and the RCCSD(T)/CBS[Q5] calculations for all orientations.

To further verify the quality of the fit we compute the relative error ∆E(%) between the original ab initio RCCSD(T)/CBS[Q5] and the values of the RKHS potential for all orientations. The results are presented in Table 3. We note that the relative error does not exceed 0.175%.

**Figure 3:**
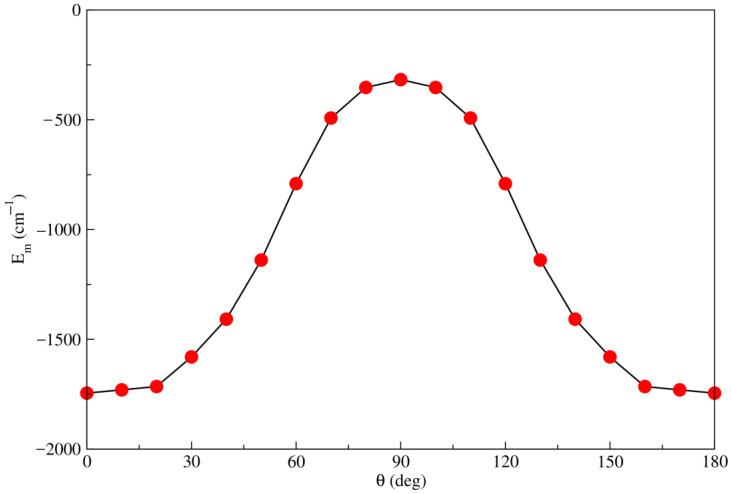
Minimum energy values obtained from the RKHS analytical interpolation with the corresponding RCCSD(T)/CBS[Q5] interaction energies as a function of angular orientations θ.

The angular minimum energy path for all configurations of the ${Li}_{2}^{+}Kr$ complex, obtained from the RKHS and the RCCSD(T)/CBS[Q5] potentials, are depicted in Figure 3. Several observations could be concluded from the behavior of the angular minimum energy path. Firstly, the RKHS fitting method demonstrates good performance in describing the ab-initio RCCSD(T)/CBS[Q5] PES for all orientations. Secondly, as the configuration changes from linear to T-shaped, the attractive effect decreases compared to the increasing potential values. Furthermore, there is a non-equally spaced energy between successive orientations, and the differences become more pronounced at angles between 30° and 60°. This phenomenon can be attributed to the emergence of repulsive effects, which become more important than the attractive ones as the Kr atom approaches the center of mass of the ionic dimer ${Li}_{2}^{+}$.

**Figure 4:**
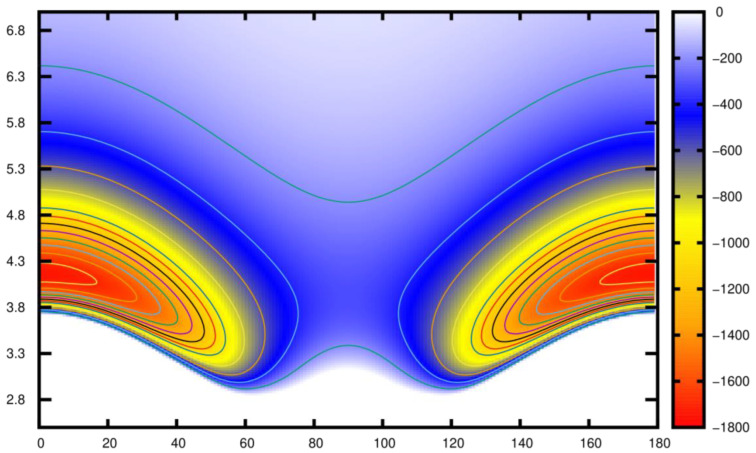
Two-dimensional contour plot of the analytical potential using the RKHS analytical interpolation in the (θ, R) plane.

Figure 4 displays a two-dimensional contour plot in the (θ, R) plane, representing the fitted RCCSD(T)/CBS[Q5] potential of ${Li}_{2}^{+}Kr$. In this plot, we observe the presence of two symmetrical minima, which correspond to the linear orientations. These minima have a well depth of 1746 cm⁻^1^ located at an internuclear distance R = 4.17 Å.

Based on the parity of the quantum number j and the total wave function, with respect to the exchange of the two identical ${Li}^{+}$ atoms, the results will be divided into two types (even/odd). It is expected to obtain a significant number of bound states when we consider the energy of the linear arrangement, 1746 cm^−1^. In our calculations, we obtained 272/260 states for even and odd symmetry, respectively. In Table 4, we list only the energies of the lowest bound states and some selected states that lie below, around, and above the T-shaped 90° barrier well for both even and odd symmetry. The other values are reported in the Supplementary Materials.

From the full table, we observe that up to *n* = 78, the even and odd vibrational states are fully degenerated. However, the higher-lying even and odd parity states exhibit different energies, and the energy difference becomes more pronounced for levels above the potential barrier in the T-shaped configuration. Furthermore, Table 4 shows that the energy of the lowest vibrational level is −1666 cm^−1^, with a zero-point energy (ZPE) of about 88 cm^−1^, which corresponds to only 5% of the well depth.

**Figure 5:**
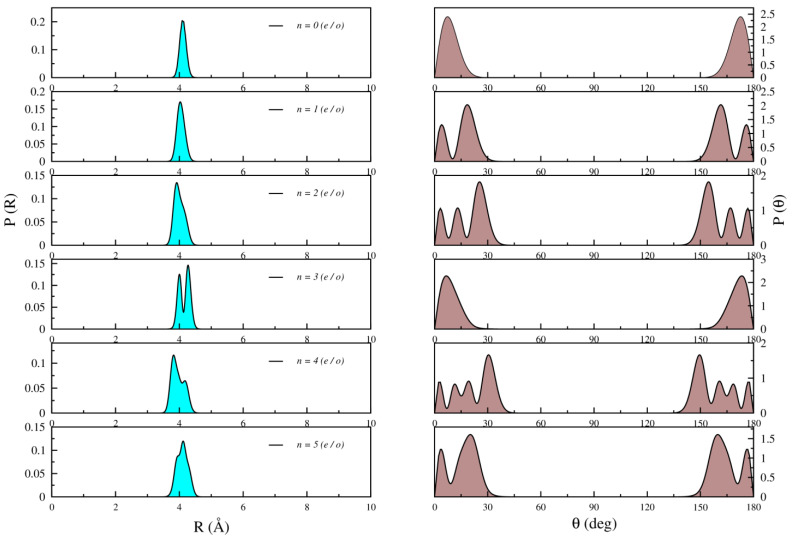
Radial (**left**) and angular (**right**) distributions of both even (e) and odd (o) states of ${Li}_{2}^{+}Kr$ complex for *n* = 0–5.

**Figure 6:**
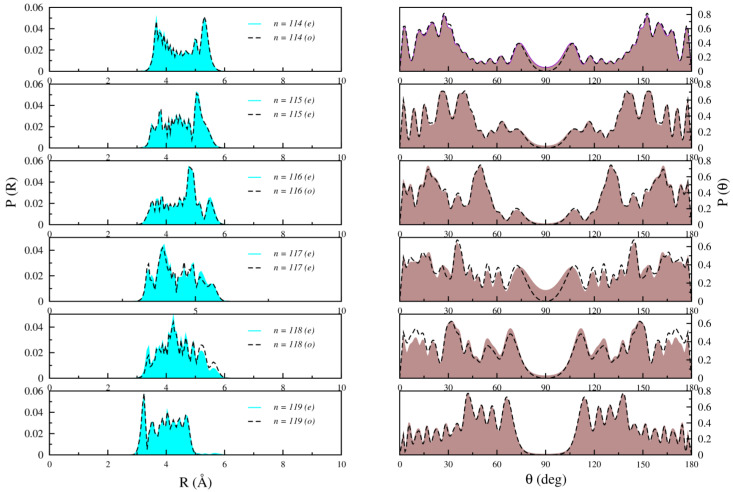
Computed radial (**left**) and angular (**right**) distributions for *n* = 114 to *n* = 119 bound states of ${Li}_{2}^{+}Kr$ for both even (right line) and odd (dashed line) states.

**Figure 7:**
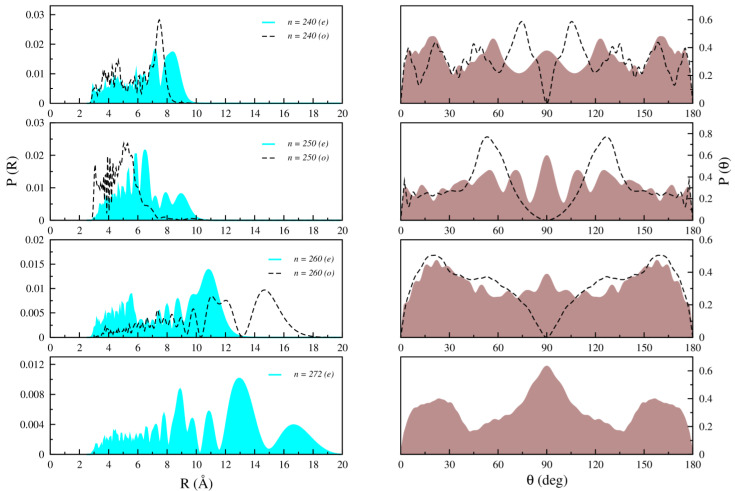
Computed radial (**left**) and angular (**right**) distributions for *n* = 240, 250, 260, and 272 bound states of ${Li}_{2}^{+}Kr$ for both even (right line) and odd (dashed line) states.

Figure 5, Figure 6 and Figure 7 illustrate the radial and angular distributions for selected states lying below, around, and above the T-shaped 90° barrier for both even and odd symmetries, respectively.

For the lowest bound states wave functions, such as *n* = 0–5, there is a minimal angular population in the region of the 90° barrier well, indicating a strong localization of the  Kr atom in the collinear geometric configuration. The ground state wave function exhibits nodal structures, with angular distributions symmetrically positioned at θ = 0° and 180°, while the radial distribution peaks at R = 4.3 Å, which is close to the equilibrium intermolecular distance of the linear geometry. The low-lying excited states can be attributed to the stretching and bending motions of the Kr atom. The bending motions correspond to the *n* = 1 and *n* = 2 levels, as evidenced by the presence of nodes in their angular distributions. The *n* = 3 state clearly displays nodes in its radial distribution, indicating stretching motions.

For the bound states that lie around the 90° barrier well (just below and above it), such as *n* = 114–119, Figure 6 illustrates more complex nodal patterns in both the radial and angular distributions. These distributions extend to larger R distances and configurations, and some population appears in the region of the T-shaped barrier. This behavior can be associated with the mixed stretch-bending vibrational “mode” of the  Kr atom along the radial and angular coordinates. Additionally, noticeable differences between even and odd states become apparent in both radial and angular probability distributions for levels *n* = 114 and above.

For the highest bound states, as depicted in Figure 7, the shape of the distributions becomes even more complicated, with extended angular distributions and prominent differences between even and odd parity states.

## 3. Computational Methods

### 3.1. Potential Energy Surface

#### 3.1.1. Ab Initio Calculations

**Figure 8:**
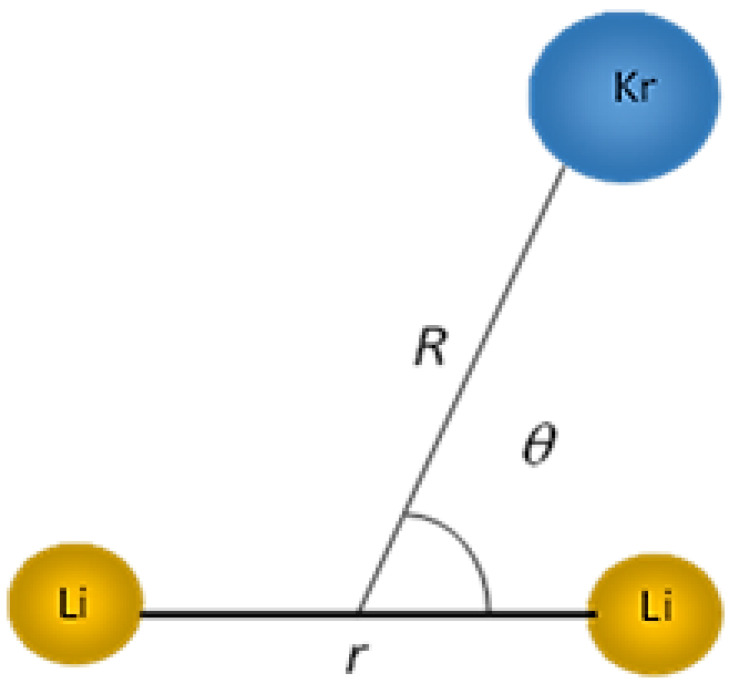
Jacobi coordinates (r, R, θ) of ${Li}_{2}^{+}Kr$ complex.

To describe the intermolecular interactions between the krypton atom and the diatomic molecule ${Li}_{2}^{+}$, Jacobi coordinates (r, R, θ) were employed, as illustrated in Figure 8. Here, r represents the equilibrium distance of ${Li}_{2}^{+}$, R denotes the distance between the center of mass of ${Li}_{2}^{+}$ and the krypton atom, and θ represents the Jacobi angle between the vectors r and R.

The calculations were carried out by keeping the diatomic ${Li}_{2}^{+}$ frozen at its experimental equilibrium distance, re = 3.11 Å, obtained via pulsed optical–optical double resonance spectroscopy [16]. The internuclear distance R was varied from 2 Å to 20 Å with an irregular step, while the Jacobi angle θ was varied from 0° to 90° with a step of 10°.

In this present work, the Molpro 2010 Package [31] was used to perform all ab initio calculations. The potential energy surface of ${Li}_{2}^{+}Kr$ was calculated using the restricted Hartree–Fock calculation followed by a single-reference restricted open-shell coupled cluster method with single, double, and no-iterative triple excitations RCCSD(T) method [32,33]. This method is known for its high level of accuracy in describing electronic correlation effects.

To assess the validity of the RCCSD(T) method employed, correlation factors ${\;D}_{1}$ and $T_{1}$ were calculated for all investigated geometries. The $T_{1}$ and ${\;D}_{1}$ diagnostics, as defined in reference [34], were used for this purpose. The $T_{1}$ diagnostic is given by $T_{1}=\sqrt{\frac{\sum_{i}^{occ}\sum_{a}^{vir}{\left(t_{i}^{a}\right)}^{2}}{n}}$, where *n* is the number of electrons, and $\left(t_{i}^{a}\right)$ represents the single excitation amplitudes. The ${\;D}_{1}$ diagnostic is defined as $D_{1}\left(CCS\right)={\left\|T\right\|}_{2}$, where ‖T‖₂ is the Euclidean norm of the matrix T  calculated from the CCSD wave function. The results of the calculations are summarized in Table 5.

The obtained results indicate that both, $T_{1}$ and $D_{1}$ factors, are below the threshold values of 0.02 and 0.025, respectively, as suggested by Lee and Taylor [34]. This confirms the suitability of the mono-configurational approach RCCSD(T) chosen for this study.

For both Li and Kr atoms, the aug-cc-pVn (*n* = 4, 5) Z basis sets [35] were utilized. The energies obtained were then extrapolated to the complete basis set (CBS) limit using a two-parameter expression [36] applied to the correlation energies as follows:$\;E_{n}=\;E_{CBS}+\frac{A}{n^{3}}$. Here, *n* = 4 and 5 correspond to AVQZ and AV5Z basis sets, respectively, $E_{n}$ is the computed total energy corresponding to that basis set, $E_{CBS}$ is the CBS extrapolated energy, and A is a fitting parameter. The energies from the AVQZ and AV5Z basis set calculations were employed for the extrapolation.

In the calculations of the internuclear interaction energies $V(r_{e},\;R,\;\theta )$ between Kr and ${Li}_{2}^{+}$, the standard counterpoise method of Boys and Bernardi [37] was used to correct the basis set superposition error (BSSE) at all configurations. This correction is applied according to the equation: $V\left(r_{e},\;R,\;\theta \right)=E_{{Li}_{2}^{+}Kr}\left(r_{e},R,\theta \right)-[E_{{Li}_{2}^{+}}(r_{e},\;R,\;\theta )+E_{Kr}(r_{e},\;R,\;\theta )]$, where $E_{{Li}_{2}^{+}Kr}(r_{e},\;R,\;\theta )$ represents the total energy of the complex, and $E_{{Li}_{2}^{+}}$($r_{e},\;R,\;\theta$) and $E_{Kr}{(r}_{e},\;R,\;\theta )$ are the energies of the ${Li}_{2}^{+}$ and Kr monomers, respectively, calculated in the full basis set of the system.

#### 3.1.2. Analytical Representation of the Ab Initio Surface

In order to conduct dynamic investigations, an analytical representation of the ab-initio potential energy surface (PES) is necessary. In this study, the 2D-PES of ${Li}_{2}^{+}Kr$ was interpolated using the reproducing kernel Hilbert space (RKHS) procedure developed by Ho and Rabitz [30]. The two-dimensional potential function is given by the following:

(1) $$V\left(r_{e},R,\theta \right)=\sum_{i=1}^{N_{R}}\sum_{j=1}^{N_{\theta }}v_{ij}q_{1}^{2,3}(R_{i},R)q_{2}(y_{j},y)$$

(2) $$V^{RKHS}\left(r_{e},R,\theta \right)=\sum_{i=1}^{N_{R}}\sum_{j=1}^{N_{\theta }}V_{ij}q_{1}^{n,m}\left(R_{i},R\right)q_{2}\left(y_{j},y\right)$$

In the above equations, y = cosθ, and $N_{R}$ and $N_{\theta }$ represent the number of calculated ab initio energies in the R and θ coordinates, respectively. The $v_{ij}$ coefficients are determined by solving Equation (1), where $V(r_{e},R_{i},\theta_{j})$ represents the ab initio RCCSD(T)/CBS[Q5] energy at each ($R_{i},\;\theta_{j},r_{e}$) grid point.

The one-dimensional distance-like $q_{1}^{n,m}$ and angle-like $q_{2}$ reproducing kernels are expressed as follows:

(3) $$q_{1}^{n,m}\left(x,x^{\prime }\right)=n^{2}x_{>}^{-\left(m+1\right)}B{\left(m+1,n\right)}_{2}F_{1}\left(-n+1,m+1;n+m+1;\frac{x_{<}}{x_{>}}\right)$$

(4) $$q_{2}\left(y,y^{\prime }\right)=\sum_{l}\frac{\left(2l+1\right)}{2}P_{l}(y)P_{l}(y^{\prime })$$

where $x_{>}$ and $x_{<}$ refer to the maximum and minimum values of x and $x^{\prime }$, respectively. $B\;{and\;}_{2}F_{1}$ represent the Beta and Gauss hypergeometric functions [38], respectively. $P_{l}$ denotes the Legendre polynomials with l = 0, 2, 4, 6, 8, 10, 12.

Based on the previous work by Alharzali et al. [12,24] for similar interactions (${Li}_{2}^{+}He$, ${Na}_{2}^{+}He$ and $K_{2}^{+}He$), it is assumed that the 2D-PES V(R, θ) of ${Li}_{2}^{+}Kr$ is a smooth function with derivatives up to the second order, *n* = 2, in both R and θ. Additionally, to account for the dominant dispersion interaction between the Kr atom and the ionic dimer ${Li}_{2}^{+}$, a weighting factor $w\left(x\right)=x^{-m}$ with m=3 is introduced.

### 3.2. Bound States Calculation

The calculations of bound states energies and wave functions are crucial steps for studying dynamics, such as vibrational predissociation and photoionization. In this study, variational quantum bound states calculations were performed using the fitted RKHS potential energies. The bound states energies were obtained by diagonalizing the Hamiltonian expressed in Jacobi coordinates, as follows:

(5) $$\hat{H}=-\frac{\hbar^{2}}{2\mu_{1}}\frac{\partial^{2}}{\partial^{2}R}+\frac{{\hat{j}}^{2}}{2\mu_{2}r_{e}^{2}}+\frac{{\hat{l}}^{2}}{2\mu_{1}R^{2}}+V(r_{e},R,\theta )$$

In the above equation, $\frac{1}{\mu_{1}}=\frac{1}{m_{Kr}}+\frac{1}{2m_{{Li}_{2}^{+}}}$ and $\frac{1}{\mu_{2}}=\frac{1}{m_{Li}}+\frac{1}{m_{Li}}$ are the reduced masses of the ${Li}_{2}^{+}Kr$ complex and the ${Li}_{2}^{+}$ dimer, respectively, where $m_{Li}$ and $m_{Kr}\;$ are the atomic masses of Li and Kr atoms. $\hat{j}$ and $\hat{l}$ represent the angular momenta associated with the vectors r (rotational momentum of the dimer) and R (orbital angular momentum), respectively. The sum of these angular momenta gives the total angular momentum $\hat{J}$, which was taken as zero in these calculations. $V(r_{e},R,\theta )$ represents the fitted 2D-RKHS potential, where rₑ is the fixed equilibrium bond length of the diatomic ${Li}_{2}^{+}$.

In the framework of zero total angular momentum J=0, a product of radial and angular basis functions is used to represent the Hamiltonian. For the angular coordinate, the employed basis function is an orthonormalized Legendre polynomial $P_{j}cos(\theta )$, where *j* ranges up to 40 for even and odd symmetry, respectively. For the radial coordinate, a discrete variable representation (DVR) basis set, based on particle in a box eigenfunctions [39], was employed. A 300 points DVR was used over a range from R = 2.5 to 20 Å, and a convergence criterion of $10^{-6}$ was established.

## 4. Conclusions

In this study, we conducted structural and dynamic investigations of the lithium cationic dimer, ${Li}_{2}^{+}$, interacting with the Kr atom. The two-dimensional potential energy surface was computed using the RCCSD(T) method and the aug-cc-pVnZ (*n* = 4, 5) basis sets, and then extrapolated to its CBS[Q5] limit. Subsequently, the RKHS method was employed for numerical interpolation to generate the RCCSD(T)/CBS[Q5] analytical potentials. Remarkably, this interpolation method accurately matched the numerical curves for all orientations used to determine the two main spectroscopic constants, Re and De, for each configuration. The analysis of the results revealed that the linear configuration, where the krypton atom is linearly attached to the lithium dimer ${Li}_{2}^{+}$ is found to be more stable than the T-shaped configuration.

The fitted 2D-RKHS potential was employed to calculate the vibrational bound state energies, resulting in a significant number of bound states as expected. It is worth noting the limited contribution of the zero-point energy (ZPE), which accounts for only 5% of the well depth. Additionally, the system exhibits very large amplitude motions, both as stretching and bending modes in its lower states, but rapidly transformed into non-regular features of their wave function in the excited states.

The obtained bound states, along with the potential energy surfaces, have various applications. They can be utilized to investigate vibrational predissociation or photoionization processes of ${Li}_{2}Kr$ complex. Furthermore, an analytical fitting of the obtained 2D-PES can be employed to explore the micro-solvation process of the lithium cationic dimer in a krypton matrix. These valuable results could assist in experimental settings and help in the interpretation of observations.

## Figures and Tables

**Table 1:** Equilibrium distance (Re) and the well depth (De) for the ${Li}_{2}^{+}Kr$ using the RCCSD(T) method and different basis sets. Re are in Å and De in cm^−1^.

Basis Set	AVQZ		AV5Z		CBS[Q5]	
θ (°)	Re	De	Re	De	Re	De
0	4.22	1730	4.17	1737	4.17	1745
10	4.14	1715	4.13	1722	4.13	1730
20	4.01	1698	4.01	1706	4.01	1715
30	3.88	1564	3.87	1572	3.87	1580
40	3.69	1391	3.68	1400	3.68	1408
50	3.52	1122	3.51	1130	3.51	1139
60	3.45	775	3.45	783	3.44	791
70	3.61	479	3.60	485	3.59	492
80	3.85	340	3.83	346	3.82	353
90	3.93	304	3.92	310	3.90	317

**Table 2:** Trends of equilibrium distances (Re) and the well depths (De) for ${Li}_{2}^{+}$ ($X^{2}\sum_{g}^{+})$ alkali dimer in interaction with He, Ne and Kr rare gas atoms. Re are in Å and De in cm^−1^.

Complex	Re	De	Method/References
${Li}_{2}^{+}$($X^{2}\sum_{g}^{+})He$	3.53	342	RCCSD(T)/CBS[Q5] [24]
${Li}_{2}^{+}$($X^{2}\sum_{g}^{+})Ne$	-	700	Pseudopotential [15]
${Li}_{2}^{+}$($X^{2}\sum_{g}^{+})Kr$	4.01	1745	RCCSD(T)/CBS[Q5]

**Table 3:** The equilibrium distance (Re) and the well depth (De) of ${Li}_{2}^{+}Kr$ complex obtained with the RCCSD(T)/CBS[Q5] and the RKHS method for θ ranging from 0° to 90°.

	RCCSD(T)/CBS[Q5]		RKHS		∆E (%)
θ (°)	Re (Å)	De ( ${\mathrm{cm}}^{-1})$	Re (Å)	De ( ${\mathrm{cm}}^{-1})$	
0°	4.17	1745	4.17	1746	0.057
10°	4.13	1730	4.13	1730	0.000
20°	4.01	1715	4.03	1715	0.000
30°	3.87	1580	3.87	1581	0.063
40°	3.68	1408	3.68	1407	0.071
50°	3.51	1139	3.50	1141	0.175
60°	3.44	791	3.44	790	0.126
70°	3.59	492	3.59	492	0.000
80°	3.82	353	4.82	353	0.000
90°	3.90	317	4.90	317	0.000

**Table 4:** Vibrational energies (in cm^−1^). of the lowest and some selected bound states (J = 0) that lie below, around, and above the T-shaped 90° barrier well of the ${Li}_{2}^{+}Kr$ complex.

n	j = even/odd
0	−1633.440613/−1633.440613
1	−1562.620803/−1562.620803
2	−1492.719188/−1492.719188
3	−1481.617393/−1481.617393
4	−1422.606448/−1422.606448
5	−1405.444466/−1405.444466
6	−1352.065571/−1352.065571
7	−1339.178576/−1339.178576
8	−1331.488421/−1331.488421
9	−1281.201931/−1281.201931
10	−1263.686846/−1263.686846
114	−335.655266/−335.655351
115	−335.190171/−335.190064
116	−326.764948/−326.764891
117	−322.031270/−322.031221
118	−318.591568/−318.591555
119	−316.216834/−316.216848
240	−25.004181/−18.144948
250	−14.747134/−8.049236
260	−6.805258/−0.477206
272	−0.298997/-

**Table 5:** $T_{1}$ and ${\;D}_{1}$ diagnostics for ${Li}_{2}^{+}Kr$ complex around the equilibrium positions.

θ (°)	$T_{1}$	$D_{1}$
0°	0.00337867	0.00727516
10°	0.00346117	0.00761505
20°	0.00973038	0.0087485
30°	0.00407142	0.00994863
40°	0.00455682	0.01166247
50°	0.00510274	0.01350919
60°	0.00546915	0.01473819
70°	0.00508529	0.01356188
80°	0.00430078	0.01101894
90°	0.00399547	0.00998581

## Data Availability

The data that support the findings of this study are available from the corresponding author upon reasonable request.

## Supplementary Materials

The following supporting information can be downloaded at: https://www.mdpi.com/article/10.3390/molecules28145512/s1, Table S1: Vibrational energies (in cm^−1^) of all bound states (J = 0) of the ${Li}_{2}^{+}Kr$ complex.
