# Supplementary material for: Structural, Spectroscopic, and Dynamic Properties of Li2+(X2∑g+) in Interaction with Krypton Atom
Source: Molecules. 2023 Jul 19;28(14):5512. doi: 10.3390/molecules28145512 (PMC10385072; doi:10.3390/molecules28145512)
Supplement: Supplementary file 1 [file molecules-28-05512-s001.zip › molecules-2439410-supplementary.pdf]

Table S1. Vibrational energies (in cm^{-1}) of all bound states (J=0) of the Li_2^+-Kr complex.

n	even	odd
**0**	-1633.440613	-1633.440613
**1**	-1562.620803	-1562.620803
**2**	-1492.719188	-1492.719188
**3**	-1481.617393	-1481.617393
**4**	-1422.606448	-1422.606448
**5**	-1405.444466	-1405.444466
**6**	-1352.065571	-1352.065571
**7**	-1339.178576	-1339.178576
**8**	-1331.488421	-1331.488421
**9**	-1281.201931	-1281.201931
**10**	-1263.686846	-1263.686846
**11**	-1254.728587	-1254.728587
**12**	-1210.250153	-1210.250153
**13**	-1204.008044	-1204.008044
**14**	-1192.120505	-1192.120505
**15**	-1178.524830	-1178.524830
**16**	-1139.460703	-1139.460703
**17**	-1125.641472	-1125.641472
**18**	-1118.218611	-1118.218611
**19**	-1102.826234	-1102.826234
**20**	-1077.028183	-1077.028183
**21**	-1069.146473	-1069.146473
**22**	-1054.918231	-1054.918231
**23**	-1043.274895	-1043.274895
**24**	-1027.103302	-1027.103302
**25**	-1000.115908	-1000.115908
**26**	-995.362309	-995.362309
**27**	-983.838271	-983.838271
**28**	-969.056649	-969.056649
**29**	-957.878865	-957.878865
**30**	-951.508862	-951.508862
**31**	-932.430766	-932.430766
**32**	-923.550886	-923.550886
**33**	-911.080608	-911.080608
**34**	-894.691809	-894.691809
**35**	-876.752200	-876.752200
**36**	-875.802402	-875.802402
**37**	-866.057287	-866.057287
**38**	-853.868857	-853.868857
**39**	-846.239054	-846.239054
**40**	-838.175510	-838.175510
**41**	-820.307666	-820.307666
**42**	-807.914033	-807.914033
**43**	-800.521214	-800.521214

**44**	-798.661008	-798.661008
**45**	-783.703299	-783.703299
**46**	-767.334290	-767.334290
**47**	-764.810601	-764.810601
**48**	-746.266147	-746.266147
**49**	-745.273455	-745.273455
**50**	-741.245073	-741.245073
**51**	-731.053240	-731.053240
**52**	-724.552828	-724.552828
**53**	-713.776254	-713.776254
**54**	-698.007612	-698.007612
**55**	-693.366499	-693.366499
**56**	-682.245773	-682.245773
**57**	-671.661647	-671.661647
**58**	-667.622642	-667.622642
**59**	-662.989748	-662.989748
**60**	-648.139069	-648.139069
**61**	-645.066295	-645.066295
**62**	-644.499597	-644.499597
**63**	-635.021411	-635.021411
**64**	-622.310146	-622.310146
**65**	-618.716236	-618.716236
**66**	-601.371193	-601.371193
**67**	-597.532016	-597.532016
**68**	-596.034632	-596.034632
**69**	-580.106926	-580.106926
**70**	-575.757232	-575.757232
**71**	-572.405539	-572.405539
**72**	-570.858524	-570.858524
**73**	-557.198392	-557.198392
**74**	-553.492563	-553.492563
**75**	-550.300814	-550.300814
**76**	-539.215321	-539.215321
**77**	-532.826748	-532.826748
**78**	-523.038005	-523.038005
**79**	-520.214230	-520.214208
**80**	-509.767191	-509.767181
**81**	-507.799668	-507.799660
**82**	-495.437728	-495.437624
**83**	-494.053566	-494.053564
**84**	-492.970361	-492.970361
**85**	-482.102875	-482.102832
**86**	-479.910828	-479.910826
**87**	-469.961671	-469.961531
**88**	-469.014987	-469.014929
**89**	-462.161734	-462.161224
**90**	-451.125974	-451.125841
**91**	-448.987285	-448.987283

**92**	-441.582958	-441.582737
**93**	-437.470233	-437.468852
**94**	-434.364370	-434.363694
**95**	-426.113286	-426.111769
**96**	-417.183412	-417.183272
**97**	-414.526835	-414.526829
**98**	-410.816160	-410.813528
**99**	-408.730034	-408.724156
**100**	-404.710484	-404.706384
**101**	-398.588039	-398.586281
**102**	-391.681624	-391.681385
**103**	-384.480422	-384.444739
**104**	-378.241590	-378.237790
**105**	-375.465934	-375.460013
**106**	-373.412282	-373.405785
**107**	-370.992685	-370.965141
**108**	-365.586656	-365.583209
**109**	-356.991032	-356.830047
**110**	-350.525580	-350.497063
**111**	-345.821929	-345.768477
**112**	-343.035580	-343.008783
**113**	-339.930204	-339.926579
**114**	-336.154470	-335.655351
**115**	-335.190190	-335.190064
**116**	-326.967420	-326.764891
**117**	-322.126732	-322.031221
**118**	-319.402039	-318.591555
**119**	-316.429060	-316.216848
**120**	-310.479390	-310.449155
**121**	-309.803829	-308.399242
**122**	-302.637200	-301.889619
**123**	-300.694359	-300.690513
**124**	-297.882316	-295.657366
**125**	-293.552094	-292.613673
**126**	-288.570649	-285.648943
**127**	-285.397777	-284.812183
**128**	-279.346880	-275.821263
**129**	-276.638867	-273.531896
**130**	-271.663650	-271.656845
**131**	-268.978102	-265.945870
**132**	-264.762051	-264.036743
**133**	-264.249271	-261.423110
**134**	-259.362935	-256.299973
**135**	-257.383348	-256.035405
**136**	-256.300319	-253.296339
**137**	-249.847076	-247.851425
**138**	-247.766757	-246.068399
**139**	-246.742613	-241.392657

**140**	-244.274310	-238.508324
**141**	-236.070607	-231.028302
**142**	-234.367003	-227.855419
**143**	-228.735620	-227.360107
**144**	-227.629203	-226.188711
**145**	-226.013830	-220.872063
**146**	-221.886008	-216.045981
**147**	-215.998645	-214.398680
**148**	-215.186520	-210.708556
**149**	-211.726538	-209.980252
**150**	-210.763985	-204.589141
**151**	-208.183009	-202.967225
**152**	-204.825115	-200.820915
**153**	-203.587174	-196.299976
**154**	-198.981359	-191.399060
**155**	-196.538659	-187.720184
**156**	-190.976751	-186.381521
**157**	-186.659251	-183.260485
**158**	-185.692183	-177.699267
**159**	-182.166439	-177.067383
**160**	-178.830527	-174.447168
**161**	-177.703331	-173.051281
**162**	-174.780551	-172.076643
**163**	-173.801567	-170.459993
**164**	-170.740887	-166.465965
**165**	-168.945597	-160.312016
**166**	-165.346170	-158.986047
**167**	-163.907098	-156.532533
**168**	-161.801452	-155.485546
**169**	-159.048414	-149.826438
**170**	-155.552597	-149.158850
**171**	-154.135699	-146.351582
**172**	-150.997073	-140.318669
**173**	-147.091837	-139.565622
**174**	-140.685900	-138.772417
**175**	-140.336160	-137.604501
**176**	-139.699353	-134.212530
**177**	-138.321697	-132.007802
**178**	-137.651720	-128.651666
**179**	-133.106074	-127.414079
**180**	-132.390629	-125.535656
**181**	-130.444826	-120.026481
**182**	-126.596190	-118.828152
**183**	-125.090168	-116.719103
**184**	-121.849465	-110.966862
**185**	-120.130924	-110.889659
**186**	-117.257964	-108.692737
**187**	-112.719023	-106.132206

**188**	-110.977615	-102.858345
**189**	-109.780071	-101.471770
**190**	-107.157916	-100.536066
**191**	-104.410126	-100.170093
**192**	-103.549318	-97.861361
**193**	-101.634000	-94.325643
**194**	-100.275427	-92.665942
**195**	-99.302223	-89.756356
**196**	-95.658633	-86.592887
**197**	-94.449501	-86.343697
**198**	-91.557723	-85.180948
**199**	-89.035808	-80.658210
**200**	-87.378916	-79.883819
**201**	-86.627744	-78.364919
**202**	-85.066146	-75.632623
**203**	-83.841110	-75.423055
**204**	-80.933574	-73.518401
**205**	-77.499790	-71.019604
**206**	-75.998964	-69.321174
**207**	-75.221675	-68.810079
**208**	-74.121400	-68.497441
**209**	-72.239523	-66.027696
**210**	-71.366394	-61.798189
**211**	-70.781194	-61.453569
**212**	-67.375816	-55.922200
**213**	-66.312019	-55.481616
**214**	-64.531838	-52.436633
**215**	-61.936977	-51.701279
**216**	-59.565489	-50.509501
**217**	-56.508114	-49.204883
**218**	-55.418843	-47.597453
**219**	-52.888778	-47.482089
**220**	-52.108139	-45.565829
**221**	-49.883682	-42.435025
**222**	-48.725213	-40.210358
**223**	-48.305731	-38.564175
**224**	-47.637842	-36.478080
**225**	-46.288238	-35.901299
**226**	-44.040651	-33.896828
**227**	-42.620369	-32.180278
**228**	-40.802217	-31.763604
**229**	-40.327101	-30.855095
**230**	-39.040878	-28.590772
**231**	-37.560886	-27.949152
**232**	-35.744436	-26.029497
**233**	-34.452494	-25.812093
**234**	-32.888760	-24.868708
**235**	-30.475817	-24.441273

**236**	-29.000869	-23.005104
**237**	-28.012576	-22.186612
**238**	-26.766502	-20.869320
**239**	-25.428008	-19.303879
**240**	-25.004181	-18.144948
**241**	-23.363920	-16.517823
**242**	-23.073108	-16.244300
**243**	-20.117918	-14.594518
**244**	-19.792897	-14.174278
**245**	-19.588812	-13.324663
**246**	-18.562221	-12.330167
**247**	-17.424198	-10.695750
**248**	-17.029103	-9.533936
**249**	-16.196485	-8.227121
**250**	-14.747134	-8.049236
**251**	-13.725253	-6.775920
**252**	-12.484047	-6.287993
**253**	-12.248368	-5.963072
**254**	-11.047422	-4.950521
**255**	-10.364018	-4.394635
**256**	-9.902893	-3.659364
**257**	-9.630883	-2.556399
**258**	-8.585007	-1.418990
**259**	-8.096044	-0.935993
**260**	-6.805258	-0.477206
**261**	-6.176322	
**262**	-5.658086	
**263**	-5.193986	
**264**	-3.928161	
**265**	-3.387457	
**266**	-2.906503	
**267**	-2.283195	
**268**	-1.611066	
**269**	-0.988698	
**270**	-0.776320	
**271**	-0.433658	
**272**	-0.298997	
